# Editorial: Palliative Care in Neurology, Volume II

**DOI:** 10.3389/fneur.2022.840110

**Published:** 2022-02-16

**Authors:** David Oliver, Marianne de Visser, Raymond Voltz

**Affiliations:** ^1^Tizard Centre, University of Kent, Canterbury, United Kingdom; ^2^Department of Neurology, Amsterdam University Medical Centres, Amsterdam, Netherlands; ^3^Department of Palliative Medicine, Faculty of Medicine and University Hospital, University of Cologne, Cologne, Germany

**Keywords:** palliative care, neurology, amyotrophic lateral sclerosis (ALS), Parkinson's disease, stroke, glioma, communication, assisted dying

Palliative care for people with neurological disease has become more accepted over the years, and there is increasing literature on this area of care. The European Academy of Neurology and the European Association for Palliative Care collaborate closely together to ensure there is involvement in conferences and they developed a Consensus Statement on palliative care for people with progressive neurological disease (1). In 2020, the International Neuro-palliative Care Society started to encourage development of care across the world and in 2021 a truly international meeting was held online (2).

In 2019, Palliative care in Neurology (3) was published, and this has been widely read and referenced. A further Research Topic was opened in 2020 and a wide variety of papers were submitted, and subsequently published. This, it is hoped, will further establish the role of palliative care for neurological patients and encourage further research into this area.

There has been increasing awareness of the palliative care needs of people with Parkinson's disease (PD). Poonja et al. in a retrospective chart review show that people with PD do show variable trajectories of motor function, as measured by the Unified Parkinson's Disease Rating Scale. However, as they approached death, there was a steep increase in the scores, regardless of their previous trajectory. Older patients, over 65 years old, were also found to have shorter prognoses. These results may help in allowing clinicians to be more aware of the changes that occur over time, particularly in considering if the person is declining more quickly and approaching end of life.

Across the world stroke is a major cause of death but the role of palliative care has often been unclear. Reisinger et al. looked at the care of stroke patients, interviewing family members and looking at decision making by the professional team. Palliative care was shown to have a major role in the support of patients and families, with specialist palliative care being integrated early into care planning. Families benefitted greatly from increased communication and support. The collaboration of stroke teams with specialist palliative care services helped in the difficult consideration and discussion of treatment at the end of life, especially if there were ethical or legal issues.

Patients with glioma often do receive palliative care, although as new treatments become available the prognosis has increased and there may be the need for complex decision making about the benefits and risks of treatment (4). Guariglia et al. evaluated the coping styles of patients with malignant glioma and their family caregivers. Initially both groups showed a fighting spirit style but at recurrence they were more likely to be fatalistic. Anxiety seemed to correlate with fatalism whereas depression was associated with fighting spirit. The changes over time are important for professional teams, as that they are aware of the need for regular assessment and consideration of the adaptions and defences of patients and caregivers.

Although palliative care has been provided to people with amyotrophic lateral sclerosis [ALS, also known as motor neurone disease (MND)] for many decades and is established within many national and international guidelines (5, 6), the challenges of people with progressive bulbar palsy (PBP), who present primarily with dysarthria and/or dysphagia, are less well-known. Bublitz et al. present a small case series of patients with PBP and show that there is a high symptom burden due to issues with oral secretions and that pulmonary infection is very common at the end of life. This shows that it is very important that these issues are discussed early in the disease progression, in advance care planning (ACP), and oral secretions are managed as effectively as possible, to enable patients to maintain as good a quality of life as possible.

Patients with acute brain injury may not have been considered for palliative care but Voumard et al. have shown that there are discrepancies between the family member's assessment of patients' goal of care compared to the care that was provided-−33% of patients had the goal of care being for comfort whereas only 11% received this care and many received more aggressive treatment. Thus, many patients may be receiving unwanted aggressive treatment, although this may be justified in the very early stages following the injury when the prognosis is very uncertain. However, at 6 months 25% of families were still unaware of the patient's goals of care, showing that it is important to reassess decision making throughout care. Thus, these ongoing and repeated discussions are an important part of ACP, as part of palliative care.

Palliative care is appropriate throughout disease progression and should be available according to need, rather than diagnosis or prognosis. There is increasing discussion of the use of ACP, where people express their wishes for care at the end of life if they are no longer able to make decisions for themselves. Kurpershoek et al. interviewed people with PD and found that the majority of patients preferred their health care professional to start the discussion of ACP, particularly in the early stage of disease. They did wish to know more about the long-term impacts of PD, although this did vary between patients. Meinders et al. present their protocol of a randomised controlled trial of the PD_Pall intervention—consultation with a trained nurse who both supports ACP and care planning and the use of a Parkinson Support Plan workbook, which encourages people to look at the issue at home and document their wishes. This study will help to elucidate the use of ACP and how health care professionals may facilitate patients with PD to think and plan ahead.

How patients with neurological disease start conversations about the disease with professionals at end of life is an important area of research. Genuis et al. report on a scoping review of communication about end of life with ALS patients, which showed very limited evidence. However, there was evidence that important areas were communication skills, together with disease specific information, such as symptom management and the use of assistive devices, in facilitating these discussions. The review also showed the need for more research so that clear guidelines could be developed.

Assisted dying—euthanasia and physician-assisted suicide—are increasingly discussed and becoming more available across the world. This has been the case particularly in neurological disease, and people with diseases such as ALS are often asking, and receiving, an assisted death in areas where this has been legalised. Nuebling et al. have studied the records within a Swiss Right-to-Die organisation and found that people with PD and atypical parkinsonian disorders were overrepresented in the cases of assisted dying. At the time of application, symptoms were commonly immobility, pain, dysarthria, and dysphagia. Atypical parkinsonian patients had a higher symptom burden and were more likely to have been diagnosed with depression. Although 80% of those diagnosed with depression received antidepressants, other symptoms, such as pain, were less well-managed. As this study showed that assisted dying was sought soon after diagnosis, there is the need to provide support for patients from diagnosis—with psychological support for patients and families.

The aim of care for neurological patients is often to support them, and their families, at home. However, admission to hospital may occur, often in an emergency situation. Willert et al. have undertaken a retrospective review of admissions and have shown that the common reasons for admission are seizures, gait disturbances, disturbance of consciousness, pain, and nutritional problems. Palliative care teams were often involved after admission, but often with delays of over 24 h, and the team approach often identified unrecognised psychosocial issues. This highlights the need for early palliative care involvement so that issues can be identified and screening in the emergency department may allow the wider palliative care issues to be picked up and managed appropriately.

The care of people with neurological illness can be difficult at home, particularly if there are cognitive changes. Vaismoradi et al. have undertaken a systematic review of the literature looking at the management of medication by caregivers of people with cognitive disorders. The review showed the importance of the assessment of the patients' needs, the role of the caregivers, and the collaboration of the wider multidisciplinary care team. To ensure safe management of medication the support of the caregivers by the wider multidisciplinary team is essential.

This collection of papers aims to provide a worldwide view of the role of palliative care for people with neurological disease. This is expanding throughout the world, with increasing awareness of the needs of patients and their families. The development of services will vary, according to the services and specific aspects of every country, but the role of palliative care early in the disease progression and being provided according to need, whether physical, psychosocial, or spiritual, is becoming accepted. People with neurological disease, together with their families and carers, may encounter many complex issues and palliative care may be appropriate and helpful, allowing an improvement and maintenance of quality of life, enabling them to live as full a life as possible until they die.

## Author Contributions

DO was the main author with contributions, comments, revision, and final version agreed by all the authors. All authors contributed to the article and approved the submitted version.

## Conflict of Interest

The authors declare that the research was conducted in the absence of any commercial or financial relationships that could be construed as a potential conflict of interest.

## Publisher's Note

All claims expressed in this article are solely those of the authors and do not necessarily represent those of their affiliated organizations, or those of the publisher, the editors and the reviewers. Any product that may be evaluated in this article, or claim that may be made by its manufacturer, is not guaranteed or endorsed by the publisher.
